# Supplementary material for: Physical activity, balance, and bicycling in older adults
Source: PLoS One. 2022 Dec 8;17(12):e0273880. doi: 10.1371/journal.pone.0273880 (PMC9731420; doi:10.1371/journal.pone.0273880)
Supplement: Attachment — Submitted filename: Response to Reviewers.docx [file pone.0273880.s002.docx]

| **Reviewer #1 Comments** | **Response** |
| --- | --- |
| Ameliorating fall risk is important, and various forms of exercise are associated with reduction in fall risk. Too, a great many researchers assume that reductions in the spatial dynamics of postural sway will reduce fall risk. The authors compared recreational cyclists vs. non-cyclists, and found some differences in sway. While the motivation is strong, critical details of the study are problematic. | Thank you for your detailed review of the paper. We understand our paper is not without limitations, but we believe we can address many of the problems you identify adequately. |
| There is a high likelihood of self-selection bias. We have no evidence of causality. It seems more than likely that older adults with poor balance would choose to not ride bicycles. Thus, it is likely that the groups in the current study differed before and independent of actual bicycle usage. At minimum, this issue must be addressed, explicitly, in a revision. | We acknowledge the likely presence of selection bias and have addressed it in the study limitations. |
| What really is needed is a true experiment—that is, a study in which individuals are randomly assigned to cycling and non-cycling groups. This might be done, for example, by buying bicycles and giving them to (randomly selected) half of a sample of non-cyclists. Pre-post measures of balance, combined with data on actual bike usage (obtainable from most cell phones, nowadays) would allow the authors to make statements about cause and effect, which they cannot do now. | We agree that implementing an intervention would be ideal to compare cyclists to non-cyclists. Future research should build off of the idea of cycling being linked to better balance to design such randomized intervention for older adults. |
| The authors have analyzed only the spatial dynamics of balance. Decades of basic research have made clear that spatial dynamics (e.g., sway velocity) differ qualitatively from the temporal dynamics of movement. Abundant evidence demonstrates that a wide variety of clinical conditions are associated with changes in the temporal dynanmics of movement, often independent of changes in spatial dynamics (e.g., Lin et al., 2008; Stergiou & Decker, 2011; Yu et al., 2013). The existing data can be subjected to a new analysis of some measure of the temporal dynamics of sway (e.g., detrended fluctuation analysis, recurrence quantification analysis, sample entropy) using validated software routines that are readily available (e.g., in MatLab). I recommend new analyses be conducted, and the results added to the revision. Doing this can only enhance the contribution of the study. | Thank you for the great suggestion to add measures of temporal dynamics. We added a detrended fluctuation analysis on COP velocity, from which we extracted measures of long-term temporal correlations (short- and long-term scaling exponents and crossover points), which exhibited some interesting differences between cyclists and non-cyclists. We agree with the reviewer that this will enhance the contribution of the study. |
| **Reviewer #2 Comments** | **Response** |
| The association between cycling and falls among older adults is an interesting, and possibly a counter-intuitive one. Further research is definitely needed. A focus on older participants builds on the available literature. | Thank you for your comment and your thoughtful review of our paper. |
| Sample – people that bicycled at least once per week during non-winter months are not particularly “cyclists”. It is very low exposure – were these people more active prior to participation in the study? What were their baseline and previous physical activity levels? | Our physical activity data presented (steps and active minutes) indicate that cyclists were more active than non-cyclists. However, we did a secondary analysis to examine the frequency of a variety of types of physical activity and found that cycling (as well as stationary cycling) are correlated with balance unlike other forms of physical activity. Additionally, we posit that low-levels of cycling may be beneficial to balance which is an extension of previous research that found that daily cycling commuters had significant increases in balance. You can find our additional analysis in table 3. |
| There are some significant differences between the cycling and non-cycling group, particularly with their physical activity levels. Given that the cycling appears to be a very small part of the overall activity, it is impossible to say that the cycling has led to the differences. It could be any of the other activities. | We acknowledge this possibility and have included analysis of self-reported frequencies of various types of PA from the survey. As noted above, cycling is most commonly associated with improved balance compared to other physical activities. See Table 3 |
| A number of between group differences were found for some of the balance variables. Why some and not others if there’s a genuine effect of cycling? | We explained EO/EC in the discussion previously. Additionally, a small sample size is a limitation to differences between groups. Other measures were approaching significance, however without a larger sample significance was not found. |
| The authors propose that “a more tightly regulated postural control strategy that may relate to higher stability” but this finding differs from previous research- why? Again, this may well be due to generally higher physical activity levels among the cyclists. | We are not stating that “a more tightly regulated postural control strategy that may relate to higher stability” differs from previous research. The differences from previous research are noted prior to that in the paragraph relating to differences found between medial-lateral sway and anterior posterior sway). I edited that sentence in hopes it clarifies those statements. "The directionality of improved balance (i.e., medial-lateral sway versus anterior-posterior sway) differ from previous research" |
| While I am favourable towards the hypothesis, the analysis does not separate the cycling from other physical activity to allow the authors to suggest that “…cycling may be contributing to the increased balance rather than cyclists’ increased levels of steps” | As noted above, we conducted a secondary analysis to examine the frequency of a variety of types of physical activity and found that cycling (as well as stationary cycling) are correlated with balance unlike other forms of physical activity. See table 3. |
| Word missing in abstract “cyclists are significantly more physically ACTIVE than…” | Corrected this error in the abstract |
